# Nursing activities and quality of care: French perspectives and leadership challenges

**DOI:** 10.1590/0034-7167-2024-0206

**Published:** 2025-03-10

**Authors:** Estefania Coello Gonçalves Canedo, Carla Sílvia Neves da Nova Fernandes, Maria Narcisa da Costa Gonçalves, María Manuela Ferreira Pereira da Silva Martins

**Affiliations:** I University of Salamanca & Pontifical University of Salamanca. Salamanca, Spain; II University of Porto. Porto, Portugal; III Porto Nursing School. Porto, Portugal

**Keywords:** Delivery of Health Care, Nurse Manager, Nurses, Quality of Health Care, Total Quality Management, Cuidados de Enfermagem, Enfermagem, Garantia da Qualidade dos Cuidados de Saúde, Gestão da Qualidade Total, Liderança, Atención de Enfermería, Enfermería, Garantía de la Calidad de Atención de Salud, Gestión de la Calidad Total, Liderazgo

## Abstract

**Objectives:**

to assess nurses’ perceptions of nursing activities that contribute to quality of care in France.

**Methods:**

descriptive cross-sectional study, between February and August 2020 in France. Sociodemographic characteristics were recorded, and nurses’ perceptions were assessed using F-EPAECQC.

**Results:**

the sample comprised 125 female nurses (21-64 years); 52% had only graduate education, and 82.4% had less than 10 years in their care unit. The F-EPAECQC score averaged 75.65 points (range 24-96). Significant associations were found between management experience and quality of care perception (p > 0.012), and between “well-being and self-care” and years of professional exercise in the unit (p > 0.001). A low hospital policies commitment was revealed with a majority of “never” and “sometimes” answers at “nursing care organization”.

**Conclusions:**

the results confirmed good French nurses’ perceptions. The statistically significant association between management experience and perceptions of the quality of nursing care activities revealed the importance of this area.

## RESUMO

**Objetivos:**

avaliar perceções dos enfermeiros sobre as atividades de enfermagem que contribuem para qualidade dos cuidados na França.

**Métodos:**

estudo transversal e descritivo, realizado entre fevereiro e agosto de 2020, na França. Foram solicitadas caraterísticas sociodemográficas, e perceções dos enfermeiros foram avaliadas usando o F-EPAECQC.

**Resultados:**

a amostra foi constituída por 125 enfermeiros (21-64 anos); 52% tinham apenas formação superior; e 82,4% tinham menos de dez anos na sua unidade de cuidados. O F-EPAECQC obteve uma média de 75,65 pontos (intervalo 24-96). Foram encontradas associações significativas entre experiência de gestão e perceção da qualidade dos cuidados (p>0,012), e entre bem-estar e autocuidado e anos de exercício profissional na unidade (p>0,001). Verificou-se um baixo compromisso com as políticas hospitalares, com uma maioria de respostas “nunca” e “às vezes” na “organização dos cuidados de enfermagem”.

**Conclusões:**

os resultados confirmaram as boas perceções dos enfermeiros franceses. A associação estatisticamente significativa entre a experiência de gestão e a perceção da qualidade das atividades de cuidados de enfermagem demonstra a importância deste domínio.

## RESUMEN

**Objetivos:**

analizar las percepciones de los enfermeros sobre las actividades de enfermería que contribuyen a la calidad de los cuidados en Francia.

**Métodos:**

estudio descriptivo transversal, conducido entre febrero y agosto de 2020, en Francia. Se registraron las características sociodemográficas y se evaluaron las percepciones de los enfermeros utilizando F-EPAECQC.

**Resultados:**

la muestra estaba compuesta por 125 enfermeros (21-64 años); el 52% sólo tenía formación de postgrado; y el 82,4% llevaba menos de 10 años en su unidad asistencial. La puntuación media del F-EPAECQC fue de 75,65 puntos (rango 24-96). Se encontraron asociaciones significativas entre experiencia en gestión y percepción de calidad asistencial (p>0,012), y entre bienestar y autocuidado y años de ejercicio profesional en la unidad (p>0,001). Se observó un bajo compromiso con las políticas hospitalarias con una mayoría de respuestas “nunca” y “a veces” en “organización de los cuidados de enfermería”.

**Conclusiones:**

los resultados confirmaron una buena percepción de los profesionales de enfermería franceses. La asociación estadísticamente significativa entre la experiencia en gestión y la percepción de la calidad de las actividades de cuidados de enfermería reveló la importancia de esta área.

## INTRODUCTION

Health care environments becomes highly complex, fast-paced, and stressful, and consequently, with potential implications on the quality of care^(1)^. Nurses play a critical and important role ensuring safety and quality by monitoring clinical conditions, detecting errors, and understanding healthcare processes and system weaknesses to ensure that patients receive high quality care^(2)^. However, to effectively achieve and maintain high standards of care, nurse managers hold a pivotal position in leading and overseeing quality improvement efforts.

Quality of nursing care is characterized by multiple determinants of its origin, including the technicality of nurses’ performance as well as their interpersonal qualities. Therefore, most indicators that operationalize and assess the quality of care can be influenced by the succession of multiprofessional activities that constitute it. Although the notion of quality has evolved and progressed with the research produced^(3,4)^, and nurse leaders increasingly need to be able to assess the perception of the care provided by their collaborators’, focusing on the achievement of excellence in the nursing professional practice^(4)^.

Nurse managers and nurse leaders needs to be involved in decision making and work collaboratively with clinicians, educators, administrators, researchers, regulators, and policy makers to find solutions to issues that may need multiple levels of improvement^(5)^. This reveals the emergence of organizations in identifying their professional definitions of quality.

In French reality, as elsewhere in the world, the quality of care provision is a priority, although the country faces several bureaucratic obstacles. At the last years, health institutions have suffered many financial cuts, as they are perceived as service-producing and not as service-providing institutions, degrading the work conditions of their own professionals^(6,7)^. Work overloaded due of a lack of recruitment is the main factor impacting the quality of care provision. This reality represents a challenge not only for the French territory, but at the global level^(8)^. This degradation of work conditions’ is reflected at the nurse care provisions, at the nurse perceptions of quality of care, as well at the nurse professional image^(8)^.

Conversely, nurses are the largest professional group in any health care system, particularly at hospitals, where they play a key role in multidisciplinary teams, and patient safety, ensuring the permanent monitoring and surveillance of patients, and ensuring the quality of care^(9)^. Considering that quality has been a growing concern of health institutions, managers, and professionals, especially nurses, the importance of studies on the quality of nursing care should be highlighted. These can contribute to minimize an important gap about the nurses’ perceptions and contributions to the quality of care in health services^(10)^. Despite the importance of measuring care quality, the number of studies conducted on this topic remains limited^(7)^, particularly within the French context.

## OBJECTIVES

To assess nurses’ perceptions of nursing activities that contribute to the quality of care in the French healthcare reality, focusing specifically on the role of nurse managers.

## METHODS

### Ethical Considerations

This study was conducted in accordance with the Declaration of Helsinki (revised in 2013) and was approved by the French Hospital (20200124). Informed consent was obtained from all participants.

### Study Design

A cross-sectional and descriptive study was conducted following the STROBE reporting checklist, to identify and assess nurses’ perceptions of nurse activities that contribute to the quality of care.

### Sample Selection

The convenience sample was requested through email to nurse managers at a French Hospital. Recruitment was carried out between February and August 2020. The final sample consisted of 125 nurses from various healthcare units who voluntarily agreed to participate in the study. The inclusion criteria were as follows: being a nurse and working at the unit for six months or more.

### Data Collection

The data collection instruments were organized and sent through online survey, with the virtual Free and Informed Consent Form (FICF), composed of an explanation of the research page and a request for data authorization. Participation invitations were sent by nurse managers of the respective units.

The sociodemographic variables analyzed were gender, age, educational level, professional experience, and management experience. In the quantitative approach the variables were the dimension of the scale “Perception of Nursing Activities that Contribute to the Quality of Care” (F-EPAECQC)^(11,12,13)^.

### Psychometric Instrument

The EPAECQC instrument was developed by Martins, et al. (2016), to identify the perception of the quality of care provided by nurses at the nurse perspective^(11,12)^. The original scale includes 25 questions. The responses are likert-type and range from 1 “never”, 2 “sometimes”, 3 “very often”, to 4 “always”. The results may range from 25 to 100 points, with a higher score indicating a higher perception of quality in the care provided^(11,12)^.

The original scale included six dimensions^(11,12)^; each dimension allows the variables evaluation resulting from the perception of the quality of nursing care provided. These variables are based on the Nursing Care Quality Standards^(14,15)^, expressed by the Portuguese nurses’ association. Which allows each respondent to assess how they perceive their implementation of each activity in the different work contexts. These are expressed in the following dimensions: “patient satisfaction”, “health promotion”, “prevention of complications”, “well-being and self-care”, “functional readaptation”, and “nursing care organization”.

The “patient satisfaction” dimension, explores the professionals’ values and respect for patients throughout the care process. It analyses holistic empathy in caregiver-patient relationships and caregivers’/family involvement^(11,12,13,14,15)^.

“Health promotion” dimension, assesses the interaction with the patient, family and community, exploring the actions of health promotion in all contexts, ambulatory, hospital, formal and informal. And how the professional can provide information which produces knowledge and new skills in the receiver of this information^(11,12,13,14,15)^.

The prevention of complications” and the “well-being and self-care” dimensions, share the same activities, based on the nursing process, incorporating diagnostics (NANDA), interventions (NIC), and outcomes (NOC), as comprehensive framework for patient care process. Allowing for an individual assessment of these activities, focused on disease prevention or treatment and improvement. It also explores how the process is performed, showing scientific/technical knowledge and rigour, as well as highlighting identified problem situations that potentially impact patients’ lives^(11,12,13,14,15)^.

“Functional readaptation” dimension considers how the professional provides continuity in the care process, plan and anticipate the discharge of patients and optimise the skills of the patient, caregiver and family to manage their recovery, treatment and functional readaptation^(11,12,13,14,15)^.

Finally, at the “nursing care organization” dimension, exclusively organisational aspects are explored, but which allows’ optimisation of continuity of care, such as proficiency in records’ systems and knowledge of institutional policies^(11,12,13,14,15)^.

This scale has been applied in different studies^(4,13,16)^, as well as internationally^(11)^. The instrument was translated and validated to the French reality in a previous study resulting in 24 questions distributed over the six dimensions^(13)^.

### Statistical analysis

For statistical analysis of the data, SPSS® 25 (Statistical Package for Social Sciences) for Windows was used. Descriptive and inferential statistical analyses were conducted. Owing to the abnormal distribution of the sample, non-parametric tests were conducted. To test the hypothesis that there was an association between the variables under study, a 95% confidence interval was adopted, with a p-value <0.05.

## RESULTS

The sample consisted of 125 nurses (Table 1). Regarding the gender distribution of the participants, 90.4% (N=113) were female. The age range was 21 years, with a mean age of 40.63 years, and a standard deviation of 9.091 years. Concerning academic qualifications, most nurses had only graduate education (52%), and only one nurse held a doctoral degree (0.8%). Regarding the duration of professional practice in the unit, 82.4% of the respondents had worked for less than 10 years.

**Table 1:** Participants characterization

Variables	n	%
Gender *(N=125)*		
Male	12	9.6
Female	113	90.4
Age Groups *(N=125)*		
20-29	19	15.2
30-39	40	32
40-49	48	38.4
50-59	13	10.4
< 60	5	4
Educational Level *(N=125)*		
Bachelors’ Degree	65	52
Licensed degree	32	25.6
Masters’ degree	27	21.6
PhD	1	0.8
Years of professional experience *(N=125)*		
> 3	9	7.2
4-10	26	20.8
< 11	90	72
Management Experience **(** *N=125)*		
Without experience	83	66.4
Experience up to 9 years	31	24.8
Experience of more than 10 years	11	8.8

The F-EPAECQC includes 24 questions distributed across six dimensions and ranges between 24 and 96 points. The score obtained was 75.65 points, confirming the existence of a good perception of quality of care. A statistically significant association was found between experience in the management area and perception of the total quality of nursing care (p>0.012). Statistically significant in the “client satisfaction” (p>0.006), “health promotion” (p>0.001), and “prevention of complications” (p>0.003) dimensions. A statistically significant association between the “well-being and self-care” dimension and years of professional exercise in the unit variable (p>0.001) should also be highlighted.

The data collected reflected a mostly positive perception of the activities performed, resulting in a good quality of care delivery (Table 2). The most representative dimensions were “well-being and self-care” and “prevention of complications”, which obtained mostly positive answers. The dimension with the greatest discrepancy was “nursing care organization,” with a majority of “never” and “sometimes” answers in Q24, indicating low nurse involvement in hospital policies.

**Table 2:** Descriptive statistics of the F-EPAECQC

			Never		Sometimes		Very often		Always	
Dim	Q	Nurses...	N°	%	N°	%	N°	%	N°	%
**Patient satisfaction**	**Q1**	Show respect for the abilities, beliefs, values, and desires of individual patient while providing nursing care.	0	0,0	15	12,0	76	60,8	34	27,2
	**Q2**	Are constantly seeking to show empathy in interactions with the patient (patient’s family).	0	0,0	5	4,0	85	68,0	35	28,0
	**Q3**	Involve significant cohabitants of individual patient in the nursing care process.	0	0,0	9	7,2	62	49,6	54	43,2
**Health promotion**	**Q4**	Identify the health situation of the population and the resources of patient/family and community.	0	0,0	33	26,4	82	65,6	10	8,0
	**Q5**	Use the hospitalization time to promote healthy lifestyles.	0	0,0	27	21,6	75	60,0	23	18,4
	**Q6**	Provide information that generates cognitive learning and new abilities in the patient.	1	0,8	28	22,4	71	56,8	25	20,0
**Prevention of Complications**	**Q7**	Identify potential problems of the patient.	0	0,0	3	2,4	83	66,4	39	31,2
	**Q8**	Prescribe and perform interventions to prevent complications.	3	2,4	13	10,4	75	60,0	34	27,2
	**Q9**	Evaluate the interventions that help prevent problems or minimize undesirable effects.	1	0,8	16	12,8	72	57,6	35	28,0
	**Q10**	Show technical/scientific rigor in the implementation of nursing interventions.	0	0,0	6	4,8	59	47,2	60	48,0
	**Q11**	Refer problematic situations to other professionals, according to the social mandates.	0	0,0	10	8,0	77	61,6	38	30,4
	**Q12**	Supervise the activities that support nursing interventions and the activities they delegate.	2	1,6	19	15,2	59	47,2	45	36,0
	**Q13**	Show responsibility for the decisions they make and for the acts they perform and delegate.	0	0,0	7	5,6	51	40,8	67	53,6
**Well-being and self-care**	**Q14**	Identify patients’ problems that will help improve the patients’ well-being and daily activities.	1	0,8	11	8,8	60	48,0	53	42,4
	**Q15**	Prescribe and perform interventions that will help improve the patients’ well-being and daily activities.	2	1,6	10	8,0	73	58,4	40	32,0
	**Q16**	Evaluate the interventions that help improve the patients’ well-being and daily activities.	4	3,2	22	17,6	64	51,2	35	28,0
	**Q17**	Show technical/scientific rigor in the implementation of nursing interventions.	2	1,6	14	11,2	57	45,6	52	41,6
	**Q18**	Address problematic situations identified that will help improve the patients’ well-being and daily activities.	0	0,0	13	10,4	77	61,6	35	28,0
**Functional Readaptation**	**Q19**	Ensure continuity of nursing service provision.	0	0,0	10	8,0	30	24,0	85	68,0
	**Q20**	Plan discharge of hospitalized patients in health institutions, according to each patients’ needs and community resources.	5	4,0	24	19,2	53	42,4	42	33,6
	**Q21**	Optimize the abilities of the patient and his/her significant cohabitants to manage the prescribed therapy.	0	0,0	7	5,6	81	64,8	37	29,6
	**Q22**	Teach, instruct, and train patients for their individual adaptation and teach, instruct, and train patients for their functional readaptation.	2	1,6	17	13,6	75	60,0	31	24,8
**Nursing care Organization**	**Q23**	Know how to handle the nursing record system.	0	0,0	20	16,0	74	59,2	31	24,8
	**Q24**	Know the hospitals’ policies.	11	8,8	64	51,2	39	31,2	11	8,8

## DISCUSSION

At the present study, similar results with the precedents studies in which the EPAECQC was implemented, Portugal and Turkey, were found^(4,11,12,16)^. The sample showed’ a mostly female population, and relatively young, as in previous studies^(4,11,12,16)^. Highlight 47.2% of the sample are located in the less 39 years class. Furthermore, we found an experienced sample, with 72% of the respondents having more than 11 years of professional experience. Conversely at the Turkish study where they found a mostly sample below 10 years of experience (83.9%)^(11)^. This new generation of nurses can be a strength but needs time to be accompanied and sustained. If care institutions do not perpetuate their employees, they generate gaps in the quality of care delivery.

At the F-EPAECQC dimensions, the French surveyed nurses seemed to value the activities inherent to the “patient satisfaction” dimension. Most of the answers at this dimension was “very often” (Q1=60,8%; Q2=68%; Q3=49,6%). Highlight the “involves significant cohabitants of individual patient in the nursing care process” activity, as the most important for the French nurses (49,6% “very often” and 43,2% “always”). This dimension seems also very important at the Portuguese study becoming even more prominent at the Turkey study^(4,11,12,16)^.

Conversely, we found a poor “health promotion” dimension. Most of the answers stills at “very often” (Q4=65,6%; Q5=60%; Q6=56,8%). This situation may be a consequence of high temporary work practice in France^(17)^. Which can lead a practice oriented but not focused to the health care promotion and prevention^(17)^. At the “well-being and self-care” dimension, we found the same tendency at the “very often” answers. Report a very close results at the three studies^(11,16)^. The overall results of this dimension demonstrated a positive perception of the activities related to the implementation of well-being and self-care activities, showing the importance that professionals give to this dimension.

At the “nursing care organization” dimension, the results demonstrated a weak perception of the activities related to the “know the hospitals’ policies” activity. This reflects a low commitment to hospital policies, potentially influenced by the prevalence of temporary work practices that do not foster a sense of identity and dedication to the institutions^(17)^. Overall, it was observed that French nurses performed the activities questioned, answering mostly “very often”, without attaining the results found at the Portuguese and Turkey study in which nurses mostly answered “always” at the activities questioned^(11,16)^.

### Study limitations

Some limitations should be acknowledged. First, the sample may not represent all French nurses, and it was smaller than initially projected due to staffing challenges arising from the COVID-19 pandemics. Second, the study was limited to a single institution, which restricts the generalizability of the findings. Consequently, future research efforts should focus on examining how nurses perceive their care delivery in broader samples that include a variety of settings.

Another possible limitation is the demographic samples’ structure and characteristics. Most participants were female (90.4%), which is consistent with the predominant gender distribution in the nursing field. However, such a high proportion may not fully reflect the diversity of the entire nursing population. The high turnover may not only indicate a low level of commitment to hospital policies. Other variables, such as lack of autonomy, may also be present and should be explored in future studies. Moreover, since the instrument relies on self-reporting, political correctness responses are inevitable. This inherent bias in the sample may have impacted the results, thus requiring additional investigation.

### Contributions for Nursing Practice, Health or Public Policy

This study highlights the structural and institutional difficulties experienced in French institutions^(15)^, emphasizing the importance of implementing strategies that reinforce the systematic implementation of activities that impact the quality of care. To improve the quality of healthcare, healthcare systems should prioritize the monitoring and evaluation of nurses’ engagement in care delivery while simultaneously identifying their strengths and addressing any barriers that hinder the provision of high-quality care.

The F-EPAECQC demonstrates validity in measuring nurses’ perceptions of their activities that contribute to quality of care, particularly as assessed by nurse managers. This instrument facilitates the evaluation of nursing teams’ quality and aids in identifying areas where additional training or institutional improvements are needed. This enables nurse leaders to enhance the training opportunities available to their colleagues and to create an improved working environment.

## CONCLUSIONS

The findings resulted from the direct perception of care providers and confirmed the positive perception of the quality of their own activities. However, a lack of implications regarding the organization of nursing care seems to prevail. An overview of areas for improvement, including formative needs, disengagement, or poor care performance, was revealed. This suggests the necessity to assess not only professional performance, but also the perception that these professionals have about their own quality of care, providing timely identification of service needs.

Identifying and discussing these situations with staff members enables nurse managers to identify both individual and collective needs, to improve working conditions and quality of care. The F-EPAECQC can be an interesting tool to help nurse managers assess these perceptions. Nurse managers are called to accomplish the great task of searching strategies to address the significant challenges evident in maintaining quality standards in care delivery, ensuring ongoing support and security for their nursing staff to promote teams’ continuity.
